# Agents of change: Understanding the therapeutic processes associated with the helpfulness of therapy for mental health problems with relational agent MYLO

**DOI:** 10.1177/2055207620911580

**Published:** 2020-03-16

**Authors:** Hannah Gaffney, Warren Mansell, Sara Tai

**Affiliations:** Division of Psychology & Mental Health, School of Health Sciences, Faculty of Biology, Medicine and Health, University of Manchester, UK

**Keywords:** Mental health, artificial intelligence, psychotherapy, psychotherapeutic processes, therapy, computer-assisted, conversational agent, relational agent

## Abstract

**Objective:**

To understand the therapeutic processes associated with the helpfulness of an online relational agent intervention, Manage Your Life Online (MYLO).

**Methods:**

Fifteen participants experiencing a mental health related problem used Manage Your Life Online for 2 weeks. At follow-up, the participants each identified two helpful and two unhelpful questions posed by Manage Your Life Online within a single intervention session. Qualitative interviews were conducted and analyzed using thematic and content analysis to gain insight into the process of therapy with Manage Your Life Online.

**Results:**

MYLO appeared acceptable to participants with a range of presenting problems. Questions enabling free expression, increased awareness, and new insights were key to a helpful intervention. The findings were consistent with the core processes of therapeutic change, according to Perceptual Control Theory, a unifying theory of psychological distress. Questions that elicited intense emotions, were repetitive, confusing, or inappropriate were identified as unhelpful and were associated with disengagement or loss of faith in Manage Your Life Online.

**Conclusions:**

The findings provide insight into the likely core therapy processes experienced as helpful or hindering and outlines further ways to optimize acceptability of Manage Your Life Online.

## Introduction

One in six people in England report a common mental health problem such as anxiety or depression.^1^ Mental health problems comprise the single main source of disability and health-related economic burden globally.^2^ However, despite high prevalence, access to treatment remains problematic and demand outstrips supply.^3^ Key government policies encourage greater adoption of digital mental health interventions to increase access at a reduced cost.^4–6^ However, existing digital interventions recommended by the National Institute for Health and Care Excellence (NICE) for common mental health problems have experienced high levels of attrition, and although active telephone support seems to facilitate effectiveness, this has increased concerns about its efficiency and cost-effectiveness.^7,8^

Digital interventions that offer greater interactivity, increased choice and control over content, and applicability to a range of psychological problems have the potential to increase acceptability and efficiency.^9^ Continued and rapid advances in digital technology are now able to facilitate this vision.^10^ One way of achieving greater collaboration and flexibility in computerized interventions is through relational agents. Relational agents are software programs that simulate a conversation through text or voice,^11^ and the efficacy and acceptability of this method of intervention appears promising.^12–16^

Demonstrating the efficacy and acceptability of digital interventions is a key focus of research and remains a vital priority.^17^ However, evaluations have often neglected to demonstrate core mechanisms of action, and as such, it remains largely unclear how interventions achieve their effects.^18^ In the context of a broader paradigm shift in psychological intervention research to focus more closely on process,^19^ the mechanisms of action are of particular importance in digital interventions, as software is updated and changed rapidly. In particular, relational agent interventions rely on dynamic change to deliver flexible, acceptable interventions to users.^20,21^ Identifying mechanisms of action, including the therapeutic alliance as an agent of change, particularly in relational agents, is a top 10 research priority in digital interventions in the UK.^21^ Investigating therapeutic processes requires a detailed analysis of what happens in therapy, how it is experienced by clients, and why they find it helpful or hindering.^22–25^ Greater transparency regarding precisely how and why digital interventions achieve psychological change is likely to increase user trust, streamline interventions to their key components, and, consequently, increase reach.^18^

Transdiagnostic interventions delivered in traditional and computerized formats have been shown to provide equivalent effects to disorder specific interventions of their type^26,27^ and offer greater interactivity, flexibility and importantly, scalability. Transdiagnostic interventions are particularly suitable for the estimated 50% of service users who experience comorbidities,^28^ or problems that do not fit into pre-defined diagnostic categories.^29^

A transdiagnostic form of cognitive therapy called the Method of Levels (MOL) is differentiated from other cognitive therapies as it originates from a unifying theoretical approach known as Perceptual Control Theory (PCT).^30^ PCT posits that psychological distress is due to sustained conflict between necessary and valued goals that are essential to all living things. Conflict disrupts successful control over important experiences and when chronic, the symptoms of psychological distress may arise. Therefore, the aim of MOL therapy is to restore and increase a client’s sense of control. This is achieved through asking curious, open questions which enable clients to talk freely and sustain focus on experiences of emotional distress. Sustained exploration of a problem increases awareness of internal conflict, facilitates new perspectives of the problem, and enables reorganization at the origin of the conflict.^31^ Reorganization is the process through which problem resolution occurs.

Manage Your Life Online (MYLO) is an online relational agent developed at the University of Manchester which aims to emulate MOL therapy.^31^ MYLO has been evaluated in two feasibility trials using student samples, with promising results.^32,33^

### Aims of the study

Primarily, we aimed to examine what users found helpful or hindering about questions posed by relational agent MYLO during intervention. Greater understanding of the core processes associated with the helpfulness of MYLO will help to optimize its acceptability and helpfulness for users. We utilized a person-based, multi-method approach to closely examine the process of intervention both within and between participants.^34^ We postulated that four key mechanisms of psychological change identified in MOL (perceived control, the ability to talk freely, to maintain a focus on emotion, and gain new perspectives) would be positively associated with ratings of the helpfulness of MYLO’s questions (primary hypothesis). Therapeutic alliance factors were also included as a comparison as these have been shown to be moderately associated with clinical outcomes.^35^

## Material and methods

### Design

We conducted a multi-method, case series design, repeated over several cases. This design was theory-led and facilitated a detailed examination of client perceptions of helpfulness in a single intervention session with MYLO. Participants were granted online access to MYLO for a 2-week period. Questionnaires were completed at baseline and follow-up and qualitative interviews conducted at follow-up.

### Participants

We recruited people who self-reported a problem that was troubling them. Initially, we attempted to recruit exclusively through clinicians at a primary care mental health service. However, this proved challenging and therefore we widened recruitment to various routes including electronic advertisement in a local peer support group, through the University of Manchester research volunteering website, and the University of Manchester counseling service. The inclusion criteria were: aged 16 and over; able to converse, read, and write in English; interested in using an online intervention; and had access to a device connected to the internet. We excluded people who had current suicidal intent or persistent self-injury, were currently psychotic, had substance dependence, a known neurological or organic basis for presentation (e.g. dementia), a moderate to severe learning disability that would affect their ability to engage with the computer program, or a visual difficulty that would impair participation.

### Ethics

The study was approved by an NHS Health Research Authority Ethics Committee. We abided by the American Psychological Association (APA) Ethical Principles and Code of Conduct. Participants provided written informed consent. No treatment was withheld due to taking part in the study. Participants were provided with a list of contacts for help in a crisis and the researcher assessed risk on a weekly basis during participation. All data collected was pseudo anonymized using a participant identification number.

### Procedures

Potential participants contacted the researcher by email or telephone and were provided with the Participant Information Sheet (PIS) via email. Eligibility was assessed verbally over the telephone using the inclusion and exclusion criteria by the researcher. If eligible, an initial appointment in a private room at the study center (The University of Manchester) was arranged by the researcher to gain informed consent, provide online access to MYLO (using a unique username and password), and complete baseline measures. Participants were advised to use MYLO at least once over 2-weeks but no upper limits on usage were applied. MYLO conversation files were screened for risk information at least weekly. Participants received a phone call mid-intervention (after 1 week) to check for any technical problems and assess risk. After 2 weeks, participants attended the study center in person to complete follow-up measures. At this session, participants were asked to read their longest MYLO conversation transcript (up to the first 30 minutes). Consistent with our primary aim to identify helpful or hindering processes in therapy with MYLO, participants were asked to identify two questions MYLO asked that they found helpful and two that they found more unhelpful. For each question identified, an intervention process measure (see measures section for detail) was completed to provide quantitative data on how helpful each question was and the extent to which the question facilitated processes identified in the literature as potentially important to psychological change. Furthermore, participants were then interviewed about the content and process of therapy with MYLO to gain deeper understanding of why questions had been identified as particularly helpful or unhelpful. Participants received £5 per completed assessment (£10 in total for both assessments).

### Intervention (MYLO)

**Figure 1.**
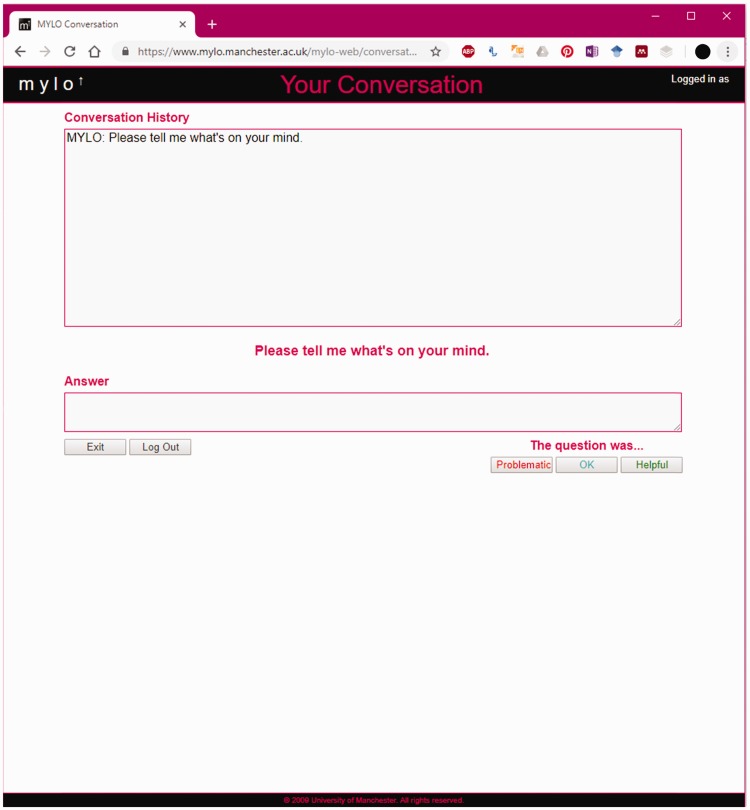
MYLO conversation screen example.

MYLO is an automated relational agent designed to deliver an MOL informed intervention through the format of text-based conversation without the support of a human clinician or therapist. MYLO is accessed online and can be used on any device through a web browser (see Figure 1) and is therefore an Internet-Operated Therapeutic Software, according to the classification of internet-supported therapeutic interventions provided Barak and colleagues.^36^ Participants were provided with a randomly generated, unique username and password to login to MYLO. Users type free-text about their problem and MYLO analyses text input for key terms, phrases, and themes. MYLO responds with questions aimed at encouraging higher-level awareness of a problem. Users can also provide real-time feedback about the helpfulness of questions to MYLO while submitting their answers. The researcher gave a short demonstration of the program at the baseline assessment and printed access instructions as a reminder. Participants were granted online access to MYLO over a two-week period and were asked to use MYLO a minimum of one time with no upper limit on usage and no suggested duration of session or frequency of use. Participants were provided with a printed list of contacts to use in a crisis.

### Measures

#### Intervention process measure

The primary outcome measure was a modified therapy process questionnaire developed from a previous MOL therapy process study.^37^ The measure retrospectively captured the therapeutic process from a client perspective in a single intervention session. The measure consisted of a rating of question helpfulness (0, not helpful at all, to 10, extremely helpful), which formed our primary outcome variable; four process items measuring key mechanisms of psychological change according to MOL, specifically, the degree to which the question enabled (a) a sense of control over what was happening in the conversation, (b) the ability to talk freely about the problem, (c) the ability to experience emotion connected to the problem, and (d) the ability to see the problem in a new way (0, not able at all, to 10, entirely able). Three therapeutic alliance items adapted from the (Session Rating Scale; SRS)^38^ were also included and measured the degree the question allowed them to: (a) feel understood and respected, (b) talk about what they wanted, and (c) the extent to which they felt the question was a good fit. All items were scored on an 11-point Likert scale. Four intervention process measures were completed by each participant for each of the questions they identified as either helpful (2 questions) or unhelpful (2 questions) from their longest conversation with MYLO.

We also used a secondary process measure called the Reorganization of Conflict scale (ROC).^39^ The ROC has three subscales—“inflexible or urgent problem solving,” “goal conflict awareness,” and “goal conflict reorganization”—and several studies have evaluated its psychometric properties.^40,41^ We used the latter 11-item sub-scale of this questionnaire, which measured the capacity for goal conflict reorganization, which is a key mechanism of change in MOL as only this sub-scale has been shown to have good internal consistency: Cronbach’s alpha 0.83.^33^

#### Intervention engagement

The frequency (total number of logins to MYLO with a conversation of any duration) and duration (total length of time in minutes of conversation, rounded to the nearest minute) of MYLO conversations were extracted using the automatic date and timestamps of conversations recorded in the MYLO program and were used as a proxy measure of engagement.

#### Symptom measures

Secondary outcome measures included the Patient Health Questionnaire (PHQ-9),^42^ the Generalized Anxiety Disorder Questionnaire (GAD-7),^43^ and the Psychological Outcome Profiles (PSYCHLOPS).^44^ The PHQ-9 is a 9-item measure of depressive symptoms with scores from 0 to 27, with a threshold score of 10 indicating clinical intervention. The measure has good internal consistency; Cronbach’s alpha 0.89.^42^ The GAD-7 is a seven-item measure of anxiety with scores from 0 to 21, with a threshold of 8 indicating clinical intervention. The measure has good internal consistency: Cronbach’s alpha 0.92.^43^ Finally, the PSYCHLOPS is a four-question, person-centered outcome measure with scores of 0 to 20. This measure assesses wellbeing, functioning, and distress. It has good internal consistency with Cronbach’s alpha 0.79 (pre-therapy) and 0.87 (post-therapy).^45^ The change score between baseline and follow-up was used to measure intra-personal change as defined by the participant.

### Qualitative interviews

Two semi-structured interviews were conducted and audio taped by the lead researcher (HG). The first interview captured participants’ subjective experiences of why they chose each of the four questions as particularly helpful or unhelpful. The topic guide outlined two main questions: (a) what made you choose that question as particularly helpful or unhelpful? and (b) what was happening in that moment? Suggestions for prompts were also outlined in the topic guide.

The second interview captured participants general views of MYLO, and feedback on the interface, usability, and design, was also gathered. The topic guide outlined questions and suitable prompts including: how easy was it for you to access MYLO; what did you think of the design of MYLO; what was your experience of putting your difficulties in writing; do you have any suggestions on how MYLO may be improved; and would you recommend MYLO to a friend? Suggestions from participants regarding enhancements or modification of MYLO were extracted to inform clear recommendations for the development of the MYLO programme.

### Statistical analysis

Analyses were performed in Stata version 15.1^46^ with an alpha level for significance of 5%. All variables were assessed for normality via histogram or boxplot inspection and skewness and kurtosis (high kurtosis is indicative of the presence of outliers). Descriptive statistics were used to describe the data. Power analysis (conducted in G*Power^47^) indicated that a total of 25 participants would be required to estimate a regression coefficient of 0.5 (a large effect) between the seven process variables and helpfulness score with 80% power at a significance level of 0.05.

#### Primary quantitative analysis

**Figure 2.**
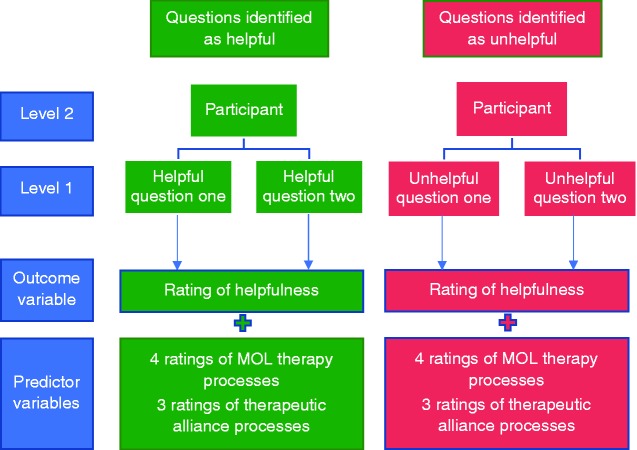
Illustration of the two-level hierarchical structure of data, split by questions identified as helpful and unhelpful.

We conducted separate analyses for questions classed as helpful and questions classed as unhelpful. The data had a two-level hierarchical structure (question process measure, level-1, and participant, level-2) which violates the assumption of independent observations (see Figure 2). Therefore, a two-level mixed effects model (STATA command MIXED) was fitted to investigate what process variables were associated with the perceived helpfulness of MYLO questions.^48^ The participant variable was entered as a random factor to account for the clustered nature of the data.

Initially, separate two-level univariate mixed effects models were fitted with the helpfulness rating as the outcome variable and each process item score as a predictor variable. Following this, a multivariate mixed effects model was fitted with the helpfulness rating as the outcome variable and all the process scores as predictors. We did not require techniques for missing data as the primary analyses were conducted only on participants who provided follow-up data. To assess the normality of the distribution of outcome variables, post-estimation residuals were plotted using histograms. Because normality assumptions were violated, the analysis was conducted again with bootstrapping (1000 iterations) to correct standard errors (SE) and provide a more accurate estimate of the confidence interval (CI) in line with guidance.^49^ This paper only reports results from the bootstrapped analyses.

#### Secondary quantitative analyses

Descriptive statistics were used to describe engagement (frequency and duration of conversations) with MYLO. Changes in psychological distress and the process of psychological organization were explored using paired sampled t-tests on scores on the ROC, PHQ9, GAD7, and PSYCHLOPS between baseline and follow-up. The study was not powered to detect significant effects; however, we report standardized effect sizes (Cohen *d*) to enable comparison with other work and future studies.

### Qualitative analysis

Interview recordings were transcribed verbatim. The intervention process interview was analyzed using thematic analysis.^50^ Thematic analysis is a flexible approach to analysis that provides a rich account and interpretation of the data.^50^ Initially, interview transcripts were read and re-read by the researcher to become familiar with the data. The first author conducted the coding inductively and themes reflecting participants’ accounts were identified. Quotes were then selected that represented each theme and themes were checked back with the transcripts to ensure they were representative of the data. This process is in line with guidance on conducting a thematic analysis^50^.

Inductive content analysis^51^ was employed to analyze general feedback from participants on their experiences of using MYLO and the design and function of MYLO. Content analysis is a replicable approach to describing and quantifying data which enables new insights and can facilitate the practical application of findings.^51,52^ This approach enabled clear recommendations for the development of MYLO. Following familiarization with the data, concepts were developed from the data and the frequencies of these between participant interviews were counted.

## Results

### Participant characteristics

**Figure 3.**
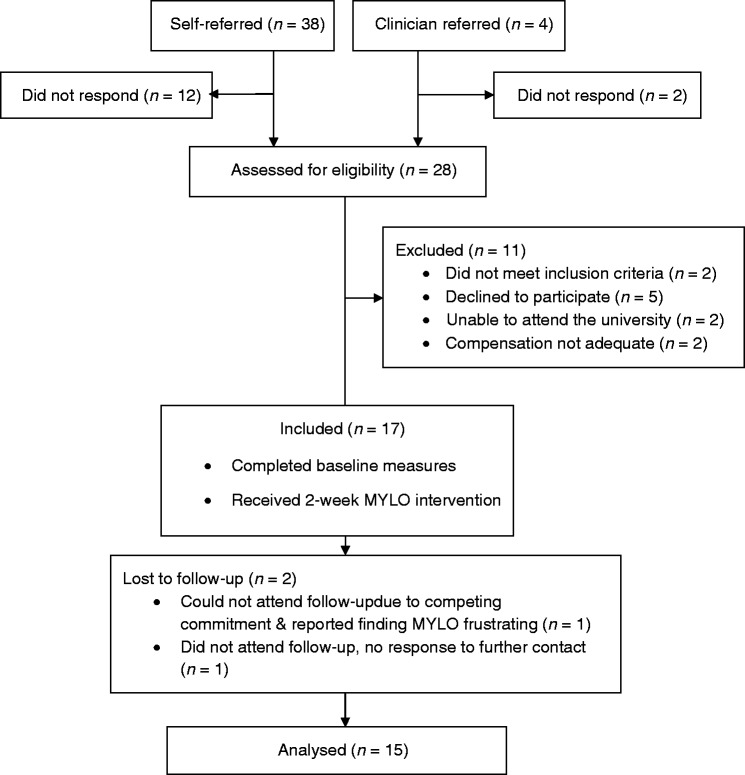
Recruitment flow diagram.

In a one-month period (November to December 2018) 28 people were assessed for eligibility. A total of 17 (60.7%) people were eligible and consented to the study and 15 completed follow-up measures (88%; see Figure 3 for recruitment flow diagram). All included participants had self-referred to the study. Due to the challenges experienced during recruitment and time constraints, we did not recruit the intended number of participants (*n* = 25).

On average, participants were aged 33.4 years (Standard Deviation; SD, 14.5) and ranged between 22 and 67 years old. There were more female (*n* = 11, 64.7%) than male participants (*n* = 6, 35.3%). Around half of the sample (52.9%, *n* = 9) scored above the clinical cut-off on the PHQ-9 at baseline and 58.8% (*n* = 10) scored above the clinical cut-off on the GAD-7 at baseline. Participants self-reported a wide range of presenting problems such as anxiety, social anxiety, depression, loneliness, bereavement, low self-esteem, concerns about work, worry, sleep problems, relationship problems, financial concerns, and career problems.

### Primary results—intervention process

#### Identification of helpful and unhelpful questions

Each of the 15 participants who attended follow-up identified two questions that they found helpful and two questions that they found unhelpful from their longest MYLO conversation transcript (See Table 1). Generally, the questions that participants appeared to find more helpful were those that picked out key words, e.g., “You used a phrase then – vicious circle – what led you to put it that way exactly?”; focused on feelings, e.g., “How does feeling confused or uncertain affect you?”; and weighed up or analyzed meaning, e.g., “What makes that important for you?” Participants identified questions that asked for elaboration with no direction or interpretation as being more unhelpful, e.g., “Please tell me a bit more by writing a few more sentences if you can.” The use of the words “mind,” e.g., “What’s coming into your mind at the moment?,” and “picture,” e.g., “If you have a picture in your mind, what is going on in it?,” appeared to be more unhelpful for participants. Interestingly, one question, “What’s running through the back of your mind while you’re typing?,” was identified more frequently than others (*n* = 8, 13%) and was classified on an equal number of occasions as both helpful and unhelpful. This is consistent with research demonstrating that client preference influences whether they experience a therapist approach as helpful or hindering.^53^

**Table 1.** Frequency table of questions identified as helpful or unhelpful.

	Helpful	Unhelpful	Total
Questions identified only as helpful			
Could you tell me what you think is the most important thing from what you are saying here?	1	0	1
How does feeling confused or uncertain affect you?	1	0	1
How does feeling uncertain affect the way you think?	1	0	1
It sounds like you are saying negative things about yourself. What makes you do that?	1	0	1
Please tell me what's on your mind (standard opening question)	1	0	1
Tell me something about why you said desperate?	1	0	1
This sounds like a sign of progress. What do you make of that right now?	1	0	1
What do you think about feeling anxious?	1	0	1
What is your experience of these memories at the moment?	1	0	1
What makes that important for you?	1	0	1
What makes you think about the idea of some kind of break?	1	0	1
What makes you use the word tired?	1	0	1
What options have you considered so far?	1	0	1
When you consider the way you think about this, how sure are you that this is how you really feel?	1	0	1
You said priorities. Can you tell me more about the alternatives you are weighing up?	1	0	1
You used a phrase then—vicious circle—what led you to put it that way exactly?	1	0	1
Questions identified only as unhelpful			
How does picturing the future like that make you feel now?	0	1	1
If you have a picture in your mind, what is going on in it?	0	1	1
Please tell me a bit more by writing a few more sentences if you can	0	2	2
Please tell me more about how you get this feeling?	0	2	2
Please try to give me a bit more detail by writing a few more sentences about it	0	2	2
Tell me something about why you said irrational?	0	1	1
What is going through your mind right now?	0	2	2
What is it about this sense you get that could be important?	0	1	1
What makes it helpful?	0	1	1
What makes you want to talk about that right now? How is it important for you?	0	1	1
What's coming into your mind at the moment?	0	3	3
What's going through your mind right now?	0	1	1
Questions identified as both helpful and *unhelpful*			
How is it to picture the future like that just now?	1	2	3
If you read back what you have just typed, what springs to mind about what you have written?	2	1	3
What do you notice about what you have just typed?	2	2	4
What makes this important to you?	1	1	2
What makes you use the word worried?	2	1	3
What would benefit you from being able to do this?	2	1	3
What's running through the back of your mind while you're typing?	4	4	8

#### Quantitative intervention process results

As described above, a rating of helpfulness (0, not helpful at all, to 10, extremely helpful) was provided for questions chosen by participants as both helpful and unhelpful. For questions identified as helpful, helpfulness scores were positively skewed. For questions identified as unhelpful, helpfulness scores were normally distributed. However, one participant rated questions they identified as unhelpful very highly for helpfulness which appeared contradictory. This participant’s qualitative interview data revealed mixed feelings about one of the unhelpful questions, “I thought the question was worded very well because it said if you can, so, it’s kind of giving you a get out clause, but then again, for me, it, it wasn’t kind of, as liberating as the, the ones at the beginning,” (Participant 33). Considering this, we used the median and interquartile range to describe the process level data as this provides a more accurate estimate of the average for data with extreme values.^54,55^ We also conducted a sensitivity analysis with this participant’s scores removed. The results of the sensitivity analysis were consistent with the primary results and therefore we report the results with this participant’s scores included.

**Figure 4.**
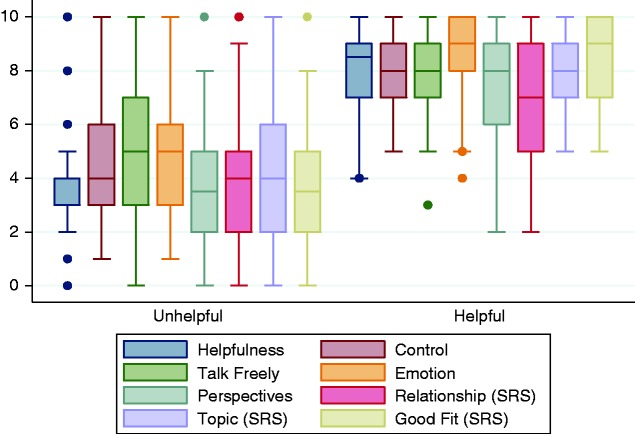
Boxplot of scores on all variables by unhelpful and helpful questions.

Descriptive statistics and distributions of scores on all intervention process variables from the intervention process measure split by helpful and unhelpful questions can be observed in Figure 4. Participants completed 32 items (eight items for four of MYLO’s questions), providing a total of 60 participant observations (30 for helpful questions and 30 for unhelpful questions) for the process analysis (see Figure 2 for an illustration of data structure).

#### Helpful questions

Seven, separate, two-level mixed effects regression models examining the association between each intervention process measure and helpfulness scores for questions classed as helpful were conducted (See Table 2). Questions that provided a greater sense of control, a sense of being understood and respected (relationship), and questions that were rated a good fit with the individual were positively associated with ratings of helpfulness for questions classed as helpful.

**Table 2.** Seven separate regression models examining the effect of each process variable on helpfulness for questions identified as helpful.

Predictor	β coefficient	SE^a^	z	*p*	Confidence interval (95%)
Control	0.51	0.22	2.28	0.022*	0.07 to 0.95
Talk freely	0.30	0.34	0.90	0.370	−0.36 to 0.96
Emotion	0.34	0.32	1.07	0.284	−0.28 to 0.96
Perspectives	0.20	0.17	1.17	0.241	−0.14 to 0.54
Relationship (SRS)	0.35	0.15	2.37	0.018*	0.06 to 0.64
Topic (SRS)	0.38	0.26	1.44	0.150	−0.14 to 0.89
Good fit (SRS)	0.82	0.13	6.24	0.000**	0.56 to 1.07

*Significant at *p* < 0.05; **Significant at p < 0.001; ^a^Bootstrapped Standard Error.

To assess the contributions made by all intervention process measures on the helpfulness of MYLO’s questions for the questions classified as helpful, a multivariate mixed effects regression analysis was conducted (See Table 3). Questions that were rated as being a good fit remained significantly associated with helpfulness. A one-unit increase in good fit rating accounted for a 0.8-unit increase in helpfulness. All other process factors were non-significant and beta coefficients were reduced compared to univariate analyses, indicating that process factors were not independent of one another (multicollinearity).

**Table 3.** Results of a multivariate mixed effects regression analysis examining the effect of all intervention process measures on perceived helpfulness for questions classed as helpful.

Predictor	β coefficient	SE^a^	z	*p*	CI (95%)
Control	0.25	0.24	1.04	0.298	−0.22 to 0.72
Talk freely	−0.16	0.30	−0.52	0.601	−0.75 to 0.43
Emotion	−0.07	0.23	−0.31	0.756	−0.54 to 0.39
Perspectives	0.08	0.14	0.57	0.566	−0.20 to 0.36
Relationship (SRS)	−0.12	0.22	−0.50	0.620	−0.53 to 0.32
Topic (SRS)	0.01	0.30	0.03	0.978	−0.57 to 0.59
Good fit (SRS)	0.86	0.37	2.33	0.020^*^	0.14 to 1.59

*Significant at *p* < 0.05; ^a^Bootstrapped Standard Error.

#### Unhelpful questions

Seven, separate, two-level mixed effects regression models examining the association between each intervention process measure and helpfulness scores for questions classed as unhelpful were conducted (See Table 4). When each process factor was entered separately, ratings of helpfulness were *positively* associated with each process factor for questions classed as unhelpful.

**Table 4.** Seven separate regression models examining the effect of each process variable on helpfulness for questions identified as unhelpful.

Predictor	β coefficient	SE^a^	z	*p*	CI (95%)
Control	0.54	0.27	1.97	0.049*	0.00 to 1.07
Talk freely	0.48	0.15	3.23	0.001*	0.19 to 0.78
Emotion	0.55	0.14	3.99	0.000**	0.28 to 0.82
Perspectives	0.50	0.16	3.17	0.002*	0.19 to 0.81
Relationship (SRS)	0.58	0.14	4.20	0.000**	0.31 to 0.85
Topic (SRS)	0.49	0.18	2.73	0.006*	0.14 to 0.84
Good fit (SRS)	0.70	0.14	5.06	0.000**	0.43 to 0.97

*Significant at *p* < 0.05; **Significant at *p* < 0.001; ^a^Bootstrapped Standard Error.

As above, to assess the contributions made by all intervention process measures on the helpfulness of MYLO’s questions for questions classified as unhelpful, a multivariate mixed effects regression analysis was conducted (See Table 5). No significant associations between process factors and helpfulness were found for questions rated as unhelpful. Beta coefficients were all reduced compared to univariate analyses indicating that process factors may not be independent of one another (multicollinearity).

**Table 5.** Results of a multivariate mixed effects regression analysis examining the effect of all intervention process measures on perceived helpfulness for questions classed as unhelpful.

Predictor	β coefficient	SE^a^	z	*p*	CI (95%)
Control	0.10	0.25	0.39	0.695	−0.39 to 0.58
Talk freely	0.02	0.26	0.08	0.938	−0.48 to 0.52
Emotion	0.28	0.24	1.17	0.243	−0.19 to 0.76
Perspectives	0.04	0.27	0.13	0.894	−0.50 to 0.57
Relationship (SRS)	0.21	0.41	0.50	0.614	−0.60 to 1.02
Topic (SRS)	−0.02	0.29	−0.07	0.94	−0.59 to 0.55
Good fit (SRS)	0.18	0.63	0.28	0.78	−1.06 to 1.41

^a^Bootstrapped Standard Error.

#### Qualitative intervention process results

Participants (*n* = 15) provided feedback on their experiences of using MYLO to explore their problems and this was analyzed using content analysis.^51^ Generally, MYLO appeared acceptable to participants and enabled participants to gain greater awareness of their problem and develop new perspectives:

… I think that it’s, more about, you know, uncovering the things that you don’t normally think about, within your own, er, mind, within your own kind of consciousness, and then helping you find your actual path to, resolving some of your issues, that you know, you might be having, and I think that it’s quite useful, especially today when, you know the mental health services are so over crowded … I think, think this is, appropriate for I think people like me, who just need to think about things from a different perspective. (Participant 20)

I think by, looking at it deeper, it’s almost like, when you tell somebody about a problem and their response is, why? and then you come back and tell them why and their response then is, why? it’s a good exercise, why? why? why? and, you, you eventually have to get to a source, it, it’s a bit like that really. (Participant 1)

it did it’s job in a way, ‘cos it made me elaborate on things and actually get to the, the root problem. (Participant 42)

Some participants appeared to build a relationship with MYLO. For example, two participants inferred feelings to MYLO e.g. “… because it’s giving those dynamic, replies, so I kind of feel like, yeah, ok, even though it’s a robot maybe it would actually be feeling sad, and sorry, kind of thing” (Participant 12) and

it seemed, interested in me, kind of, and without, being kind of sympathetic, overly sympathetic or, it, it wasn’t kind of, er, the expectations, you know and the more I felt like I could have gone with it, the more I would have got out of it, so it was kind as if I, I felt in control. (Participant 33)

However, some participants (*n* = 4) reported that they were aware of MYLO’s limitations, e.g., “I was aware all the time that there wasn’t a person, that, that became quite, apparent, the more I used it, that it was, an automated response almost.” (Participant 1). Three participants indicated that they had difficulty engaging with MYLO fully due to high distress and/or low motivation e.g. “because I was struggling a bit, I think I used it just quite sparingly, not, not a lot to be honest, as much as I would have wanted to …” (Participant 11).

Overall, when asked in interview, most participants reported that they would recommend the intervention to a friend (*n* = 12). Three participants indicated that they thought it was most suitable for people with low–moderate levels of common mental health problems such as anxiety and depression, e.g., “I think it could be quite a good tool for some people, that are having, problems with anxiety or depression, in the early stages” (Participant 1). Three participants thought it could be used alongside face-to-face therapy, e.g., “… really good, tool for, sort of complementing therapy, rather than, providing therapy” (Participant 12), and one participant highlighted the high demand for psychological services and thought it would be a useful intervention while on a waiting list for face-to-face therapy, e.g., “if they needed to talk to some, something to get, to get things out, then, yeah, ‘cos, therapy’s not easy to get, you’re on a waiting list unless you’re suicidal basically” (Participant 2).

**Figure 5.**
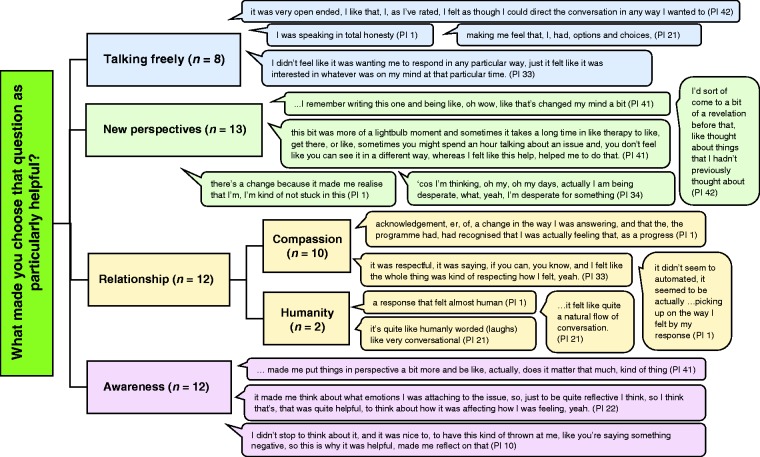
Thematic map of participants’ reasons for choosing a question as being particularly helpful.

All participants who attended follow-up (*n* = 15) were interviewed about why they had chosen questions as helpful or unhelpful. This interview was analyzed inductively using thematic analysis. Figure 5 illustrates a thematic map of participant’s responses to the question “What made you choose that question as particularly helpful?” Four major themes of talking freely, new perspectives, relationships, and awareness were identified from the qualitative data. Two subthemes of compassion and humanity were identified within the main relationship theme. Specifically, questions that enabled a sense of being able to express themselves freely and in any direction of their choosing (talking freely; *n* = 8); questions that enabled participants to begin to see their problem in new ways and gather new perspectives (new perspectives; *n* = 13); questions that encouraged greater reflection and awareness of the details and emotions attached to a problem (awareness; *n* = 12); questions that demonstrated understanding and compassion in relation to feelings (relationship subtheme; compassion; *n* = 10); and questions that felt more human and natural (relationship subtheme; humanity; *n* = 2) were associated with helpfulness.

**Figure 6.**
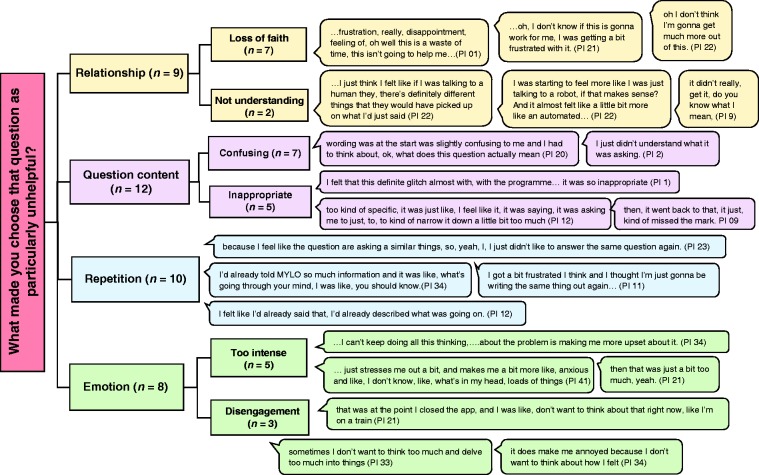
Thematic map of participants’ reasons for choosing a question as being particularly unhelpful.

Figure 6 illustrates a thematic map of participants responses to the question “What made you choose that question as particularly unhelpful?” Four major themes of relationship (subthemes: loss of faith and not understanding), question wording (subthemes: confusing and inappropriate), repetition, and emotion (subthemes: too intense and disengagement) were identified from the interviews regarding questions classed as unhelpful. Specifically, questions that revealed that MYLO had not really understood the participant (relationship; subtheme; not understanding; *n* = 2) and appeared to result in a loss of faith in MYLO more generally (relationship; subtheme; loss of faith; *n* = 7); question content or wording that was confusing (question content; subtheme; confusing; *n* = 7) or inappropriate (question content; subtheme; inappropriate; *n* = 5); questions that were repetitive or required participants to repeat things they had already stated (repetition; *n* = 10); questions that were too emotionally intense or required thinking about something that was too emotionally difficult at that time (emotion; subtheme; too intense; *n* = 5) and appeared related to disengagement with MYLO (emotion; subtheme; disengagement; *n* = 3) were associated with unhelpfulness.

### Secondary results—Engagement, design and function, and clinical outcomes

#### Engagement with MYLO

The frequency of conversations with MYLO across the two-week intervention period varied between participants from a minimum of 1 to a maximum of 6. The mean number of conversations per participant was 2.53 (SD 1.38). The time each participant spent using MYLO ranged from a minimum of 9 minutes to a maximum of 129 minutes. On average, participants spent 35.7 minutes (SD 32.45) using MYLO in the 2-week period. Generally, participants found MYLO’s questions acceptable, with 93% (238/255) of questions rated “OK” by participants, 8.6% (22/255) rated as particularly helpful, and 7.8% (20/255) rated as problematic.

#### Design and function

No participants reported any technical problems with MYLO during the intervention period. Participants reported mixed feelings about the MYLO intervention design, functions, and accessibility (see Table 6). Participants reported that MYLO was a quick, easy to access, and available intervention which was simple to use and non-judgmental. However, participants expressed that they would like MYLO to be available as an app they could download and login to independently. Participants suggested several improvements to the content, such as a greater range of questions with increased individualization (e.g., key word hits), the use of notifications to remind users to use the app, and the ability to view historical conversations. Participants also suggested several improvements to the design including modernizing the look, using a speech bubble format with automatic scrolling and including the option to create an avatar to represent themselves. Two participants indicated that they would like to see more color across the app generally.

**Table 6.** Participant feedback on MYLO intervention functions and design.

Positive feedback	Suggestions for improvement
Access	
Easy to access (*n* = 12)	Available as an app (*n* = 7)
Available (*n* = 5)	Available through search engines (*n* = 1)
Quick responses (*n* = 5)	Register for app independently (*n* = 1), i.e., generate own username and password
Private (*n* = 1)	
No judgment/reduced stigma (*n* = 1)	
Functionality	
Information about question button (*n* = 2)	Increase range of questions (*n* = 3)
Question feedback buttons (*n* = 3)	Reduce question repetition (*n* = 7)
	Increase individualization (*n* = 2)
	Provide summary/feedback of conversation at the end (*n* = 1)
	View historical conversations (*n* = 3)
	Mood rating graph (*n* = 1)
	Crisis contact information on main window (*n* = 1)
	App notifications/reminders (*n* = 2)
Design/interface	
Simplicity (*n* = 11)	Automatic scrolling (*n* = 3)
Ease of use (*n* = 2)	Speech bubble format (*n* = 4)
	Modernize (*n* = 4)
	More use of color (*n* = 2)
	Buttons instead of hyperlinks (*n* = 1)
	Avatar/picture for MYLO and user in conversation screen (*n* = 1)

#### Clinical outcomes

Differences between baseline and follow-up scores were normally distributed on all clinical outcome measures and there were no outliers. A small, non-significant increase was observed on ROC scores between baseline (72.48, SD 11.63) and follow-up (76.96, SD 12.84), t(14) = 2.02, *p* = .063, indicating some improvement in capacity for reorganization (the process through which conflict is resolved according to PCT^30^). Paired samples t-tests indicated small, but non-significant, reductions in anxiety, depression, and distress between baseline and follow-up (See Table 7), although due to the study design, the reasons for this cannot be attributed to the MYLO intervention. At follow-up, a third of the sample (29.4%, 5/15) scored above the clinical cut-off on the PHQ-9 and 41.2% (7/15) scored above the clinical cut-off on the GAD-7.

**Table 7.** Scores on clinical outcomes at baseline and follow-up and results of paired samples t-tests.

	Baseline (*n* = 15)Mean (SD^a^)	Follow-up (*n* = 15)Mean (SD)	Mean difference (95% Confidence interval)	t	df	*p*	Effect size (Cohen’s d)
PHQ-9	10.13 (5.37)	8.53 (5.58)	−1.6 (−3.27 to 0.07)	−2.05	14	0.059	0.29
GAD-7	9.00 (4.88)	8.27 (4.96)	−0.73 (−2.31 to 0.84)	−1.00	14	0.334	0.15
PSYCHLOPS	12.07 (3.17)	11.20 (4.60)	−0.87 (−2.53 to 0.79)	−1.12	14	0.282	0.22

^a^Standard Deviation

## Discussion

### Main findings

As far as we are aware, this is the first study to investigate therapeutic processes from a client-perspective, for a relational agent intervention, and utilize a cohesive theory to understand these processes.

All of the therapy process factors (control over what was happening in conversation, the ability to talk freely, the ability to experience emotion, to see the problem in a new way, to feel understood and respected, to talk about the topic they wanted, and the extent to which the question was a good fit), were consistently rated highly for questions identified as helpful (median scores ranged from 7 to 9 out of 10) and consistently lower for questions identified as unhelpful (median scores ranged from 3.5 to 5 out of 10). However, contrary to our hypothesis, only one of the process factors—“good fit”—from the SRS was significantly associated with the helpfulness of MYLO questions in multivariate analyses (see limitations section for further discussion).

However, the results of our qualitative analysis provide partial support for our hypothesis. Compassionate and human-like questions which enabled participants to talk freely, increase awareness of their problem, and gain new perspectives were identified as helpful. This is consistent with key mechanisms of change identified in MOL and PCT.^30,31^ Notably, MYLO’s therapeutic approach (curious questioning using MOL) is exclusively concerned with enabling the client to develop their own understanding of the problem to gain new insights and solutions, which is rather different to other relational agent interventions that have a greater focus on psychoeducation, advice giving, or teaching/learning new skills.^56–58^ Our findings are also consistent with research indicating that maximizing the opportunity to talk freely is important for users, e.g., through enabling free text input and tailoring the session content or duration of sessions.^58^

Repetitive, confusing or inappropriate questions, which highlighted MYLO’s lack of understanding, were associated with a loss of faith in the MYLO intervention and were identified as unhelpful. These themes are consistent with studies of other relational agent interventions^56–61^ and are perhaps not unique to MYLO. Furthermore, questions that elicited overwhelming or intense emotions were identified as unhelpful and appeared to be associated with disengagement from MYLO. This finding is supported by research suggesting there is an optimal level (a moderate amount) of emotional arousal in therapy which is associated with better outcomes.^62^

Interestingly, the overarching process of control identified in PCT as key to psychological change was not identified as related to helpfulness. However, it might be hypothesised that being “in control” is what enables a person to talk freely, experience emotions that are manageable, develop awareness, and gain new insights. Moreover, unhelpful processes such as incidences where MYLO lacked understanding or asked questions that “delved too deep” for comfort could be hypothesised as an experience of a loss of control. Future research could investigate this further perhaps by using methods such as mediation analysis.

Generally, participants found MYLO to be an accessible and acceptable intervention format which was simple to use and had the potential to provide flexible support, either as a complement to existing treatments or as a standalone. Despite significant challenges in recruiting through clinicians, upon advertising within the wider community, interest was high (all participants self-referred within a 1-month period) and drop-out rates were low (2/17, 12%). However, we recognize our intervention was short in duration. Participants reported a wide-range of presenting problems and over half of participants scored above clinical thresholds for anxiety and/or depression at baseline. No participants reported a worsening of symptoms at follow-up and small but non-significant reductions in psychological distress were found, although this was not a key aim of this study and we are cautious about drawing any conclusions from this (see limitations). Finally, several improvements to the design and functions of MYLO were highlighted, including making MYLO available as an app, modernizing the look and feel, and adding functions such as notifications and conversation history.

### Strengths and limitations

We conducted a comprehensive, multi-method analysis that examined the process of therapy with MYLO in detail, from a client centered perspective, to gain insights into what is helpful and hindering about the current MYLO intervention. We were inclusive in our entry criteria and did not exclude people based on mental health diagnosis or concurrent or previous psychological treatments. No participants reported any technical problems or problems understanding how to use MYLO.

Significant challenges to recruitment through clinicians resulted in a small, exclusively self-selected sample recruited through study adverts throughout the University of Manchester and a local peer support group. Furthermore, some participants were unable to take part due to having to travel to the University for assessments. This limits the generalizability of the findings. The challenges of recruiting through a primary care mental health service perhaps reflects the rare uptake of digital interventions in Improving Access to Psychological Therapies (IAPT) services, despite a key aim of IAPT to provide treatment to as many clients as possible.^63^ Moreover, the small sample resulted in limited statistical power and thus also limits the ability to draw firm conclusions about core intervention process especially in relation to the quantitative results. Related to this, we were unable to conduct a more robust simulation-based power calculation that would account for the hierarchical data structure as we did not have prior estimates of important parameters (e.g. from previous studies).^64^ We did not aim to demonstrate efficacy, as a much larger sample, a longer period with the intervention, and a control condition would be required to investigate this. Therefore, we cannot draw any conclusions about the effectiveness of the intervention from this study. Additionally, we did not collect data on specific mental health diagnoses, psychotropic medication, or current or previous psychological treatment; therefore, we cannot make inferences about for whom, and for what difficulties, MYLO is most suitable for or any potential interactions with other treatments.

The intervention process measure was developed and tested previously in a study of face-to-face MOL therapy^37^ and therefore may not be applicable in the same way to a digital intervention. There is little agreement in the literature as to how to measure the digital therapy process and no specific measures have yet been developed.^65,66^ The process factors were all highly correlated (multicollinearity), suggesting that these concepts may not be independent or distinct from one another. Multicollinearity limits the conclusions that can be drawn from the quantitative process analysis as the parameter estimates in the multivariate model can be biased and imprecise.^67^ However, the findings have important implications for future studies examining processes of therapy quantitatively, e.g., the importance of ensuring the accurate measurement of distinct concepts to mitigate multicollinearity problems. Asking participants to identify two helpful and two unhelpful questions from their longest conversation may have biased our results by identifying only the extremes and only provides a snapshot of moments that may not be representative of the conversations and intervention as a whole. Furthermore, participants may have had difficulties recalling experiences after a two-week interval.

### Conclusions

Importantly, the intervention appeared acceptable to participants with a wide variety of presenting problems of varying severity, which has the potential to significantly extend the applicability and reach of the intervention compared to disorder specific interventions. Despite their different presenting problems, participants identified similar processes as either helpful or hindering, providing support to the transdiagnostic model of psychological disorders,^26^ and, more specifically, the importance of transdiagnostic processes of talking freely, gaining higher level awareness, and developing new perspectives as outlined in PCT.^30^ This supports research indicating that a vital ingredient in helpful therapy is the ability to freely explore what is on your mind.^37,68,69^ Moreover, due to the dynamic nature of relational agents, each participant experienced the intervention differently depending upon which questions were posed by MYLO. Despite this, participants’ views on why questions were particularly helpful or unhelpful appeared to converge and provided insight into what clients found fundamentally important for a helpful intervention and recommendations on how to improve MYLO going forward. Our findings support the call to reconsider constraints on how therapy is delivered and, importantly, to consider core mechanisms of action over highly specified and manualized treatment protocols.^70,71^

Finally, all participants were recruited from the community. This suggests that there are a proportion of people that are not accessing services but are actively seeking psychological support. This is supported by research indicating a significant mental health treatment gap in the UK.^1,72^ Digital interventions such as MYLO may be one way to meet this unmet need and, crucially, vastly improve accessibility through avoiding the need for multiple steps including diagnosis, referral from a GP, and acceptance into a mental health service.
